# A Powerful Yeast Model to Investigate the Synergistic Interaction of α-Synuclein and Tau in Neurodegeneration

**DOI:** 10.1371/journal.pone.0055848

**Published:** 2013-02-05

**Authors:** Gianmario Ciaccioli, Ana Martins, Cátia Rodrigues, Helena Vieira, Patrícia Calado

**Affiliations:** 1 BIOALVO, Serviços Investigação e Desenvolvimento em Biotecnologia S.A., Edificio ICAT, Campus da FCUL, Campo Grande, Lisboa, Portugal; 2 DEIO and BIOFig Center, Faculty of Sciences, University of Lisbon, Lisbon, Portugal; “Mario Negri” Institute for Pharmacological Research, Italy

## Abstract

Several studies revealed consistent overlap between synucleinopathies and tauopathies, demonstrating that α-synuclein (ASYN) and tau co-localize in neurofibrillary tangles and in Lewy bodies from Alzheimer’s and Parkinson’s disease patients and corresponding animal models. Additionally, it has been shown that ASYN can act as an initiator of tau aggregation and phosphorylation and that these two proteins directly interact. Despite these evidences, the cellular pathway implicated in this synergistic interaction remains to be clarified. The aim of this study was to create a yeast model where the concomitant expression of ASYN and tau can be used to perform genome wide screenings for the identification of genes that modulate this interaction, in order to shed light into the pathological mechanism of cell dysfunction and to provide new targets for future therapeutic intervention. We started by validating the synergistic toxicity of tau and ASYN co-expression in yeast, by developing episomal and integrative strains expressing WT and mutant forms of both proteins, alone or in combination. The episomal strains showed no differences in growth delay upon expression of ASYN isoforms (WT or A53T) alone or in combination with tau 2N/4R isoforms (WT or P301L). However, in these strains, the presence of ASYN led to increased tau insolubility and correlated with increased tau phosphorylation in S396/404, which is mainly mediated by *RIM11*, the human homolog of *GSK3β* in yeast. On the other hand, the integrative strains showed a strong synergistic toxic effect upon co-expression of ASYN WT and tau WT, which was related to high levels of intracellular ASYN inclusions and increased tau phosphorylation and aggregation. Taken together, the strains described in the present study are able to mimic relevant pathogenic features involved in neurodegeneration and are powerful tools to identify potential target genes able to modulate the synergistic pathway driven by ASYN and tau interaction.

## Introduction

In the last decade, several studies, from genetic features to direct interaction, have revealed a consistent overlapping between synucleinopathies and tauopathies [1]–[3]. Synucleinopathies consists of a group of disorders in which the pathological hallmark is the presence of insoluble fibrillary aggregates of alpha-synuclein (ASYN) protein, designated by Lewy bodies, in specific brain cells populations. The disorders included in this group are Parkinson’s disease (PD), dementia with Lewy bodies (DLB) and multiple system atrophy (MSA) [4], [5]. The first two mentioned diseases are characterized by the presence of Lewy bodies in the dopaminergic neurons in the substantia nigra of the mid brain region and in the cholinergic neurons in the brainstem, limbic and cortical region, whereas in MSA glial cells are affected showing a high density glial cytoplasmatic inclusions (GCIs) ASYN-positives [4], [5]. In most cases PD is a idiopathic disease, involving polygenic mutations, gene interactions and lifestyle, whereas monogenic forms are very rare with only around 20% of cases reporting hereditary features [6]–[9]. ASYN gene (SNCA) was the first gene discovered to be associated with PD. It was observed that duplication or triplication [10], [11] of the SNCA gene, along with three missense mutations (A30P, E46K and A53T), are all related to familial PD [12]–[14].

Tauopathies represent another group of neurodegenerative disorders also characterized by the aberrant aggregation of specific proteins. The pathological hallmark of tauopathies consists in the presence of neurofibrillary tangles (NFT) labelling positive for tau protein [15], [16]. The accumulation of these tangles leads to progressive brain atrophy due to frontotemporal or striatonigral degeneration. Pathologies such as Alzheimer’s disease (AD), frontotemporal lobar degeneration (FLD), progressive supranuclear palsy (PSP) and corticobasal degeneration (CBD) are all examples of tauopathies [17]. How NFTs are formed in these diseases is still under investigation. It is known that the paired helical filaments (PHF) of NFT are constituted by hyperphosphorylated tau, mainly promoted by the kinase *GSK3β* which was shown to phosphorylate tau in up to 30 distinct sites [18]–[20]. In addition to hyperphosphorylation, there are also some tauopathies such as frontotemporal dementia with parkinsonism linked to chromosome 17 (FTDP-17) in which tau’s missense mutation P301L is known as an “aggregation-prone mutation” [21], [22].

The overlapping between synucleinopathies and tauopathies involves many aspects. Genome-wide association studies have shown that the microtubule-associated protein tau (MAPT) haplotype H1 displays a significant association with PD [3], [23]. Other studies showed ASYN being localized in NFTs, which are mainly composed by tau, and tau localized in Lewy bodies, mainly composed by ASYN [2], [24]–[26]. In fact, in a double transgenic mice expressing human ASYN WT in combination with human P301L mutant tau, these two proteins have been shown to co-localize in the same intracellular inclusions [27]. A direct binding between these two proteins has also been described with disease-related mutations being shown to interfere with this interaction [1], [28]. Moreover, it was also described that ASYN can act as a pathological initiator of tau phosphorylation and aggregation, both *in vitro* and *in vivo* [29]–[32]. Whereas reduction of endogenous tau levels in murine models of Alzheimer’s disease has been shown to improve cognitive performance [33]–[35], tau reduction did not prevent motor deficits in mouse models of PD [36]. These observations rely on the use of two specific animal models and cannot be extrapolated for other models or to the human PD condition without further validation. In addition, implication of tau levels in cognitive function cannot be ruled out. Interestingly, neurite degeneration has been directly correlated with tau protein levels and phosphorylation state in a Drosophila model of PD [37]. Therefore, the mechanism by which ASYN and tau synergistically interact and its role in pathology deserves further investigation.

Yeast models are validated tools for the study of neurodegenerative diseases [38], [39]. In fact, both cellular mechanism of action and phenotypical repercussions derived from ASYN expression have been largely studied in yeast [40], [41], a non-expensive, easy to handle and with a short replication time model. Whereas ASYN expression in yeast has been widely described as causing toxicity and growth delay, expression of tau in yeast has no apparent consequence on cell growth [42], [43]. Neither ASYN nor tau have orthologues in yeast, thus allowing the development of unbiased models of human transgene expression, with no interference or competition from endogenous yeast proteins. Co-expression of ASYN and tau was previously promoted in yeast and was reported to be synergistically toxic, although ASYN expression alone resulted in no phenotype [44]. We wanted to further explore this synergistic phenotype, by making use of distinct expression systems, where protein levels and transgene copy numbers can be more stably maintained. Our goal was to develop a yeast model of ASYN and tau co-expression that will be used for the identification of genes able to modulate this synergistic interaction and hence relevant as future therapeutic targets for human neurodegenerative diseases. Therefore, in the current work, we report the development of different wild type (WT) yeast strains, BY4741 and W303-1A, co-expressing ASYN and tau from episomal or integrative vectors, with the aim of identifying a toxic phenotype caused by the synergistic interaction between the two proteins. Both episomal and integrative strains were further characterized by analyzing the formation of ASYN intracellular inclusions and tau aggregation and phosphorylation states. This detailed characterization allowed the identification of a synergistic toxic effect probably triggered by the presence of ASYN WT cytoplasmatic inclusions and insoluble phosphorylated tau WT. The yeast strains developed in this work are being used as tools for the identification of potential target genes able to modulate the synergistic interaction observed between ASYN and tau in neurodegenerative diseases.

## Materials and Methods

### Yeast Strains and Media

The following *Saccharomyces cerevisiae* strains were used in this study: BY4741 (*MATa his3Δ1 leu2Δ0 met15Δ0 ura3Δ0*), W303-1A (*MATa leu2-3,112 trp1-1 can1-100 ura3-1 ade2-1 his3-11,15*) (T. Outeiro IMM) and the single deletion mutant ΔRIM11 was obtained from the genome-wide yeast deletion collection YSC1053 (Open Biosystems) [45]. All strains were grown in synthetic complete media (SC) which consists of 0.67% yeast nitrogen base (Sigma-Aldrich), 0.067% yeast drop-out mix (MP Bio) and 2% (w/v) carbon source glucose, galactose or raffinose, (Sigma-Aldrich) depending on the experiments, solid media plates also contain 2% agar (BD).

### Plasmids

Tau (2N/4R) WT and P301L variants were subcloned from YIpLac128 Gal1 (BIOALVO) into pESC-LEU, a bidirectional high-copy (2µ) episomal vector (Stratagene), under Gal1 promoter using the restriction sites ApaI N-terminal and XhoI C-terminal. ASYN WT and A53T variants were subcloned from pRS426 GPD (T. Outeiro IMM) into the same pESC-LEU vector but under Gal10 promoter using the restriction sites SpeI N-terminal and HindIII/SacI blunt end C-terminal and into pRS306 Gal1 (BIOALVO) using the restriction sites SpeI N-terminal and HindIII C-terminal. BY4741 WT and ΔRIM11 yeast strains were transformed with the episomal vector pESC-LEU harbouring ASYN (WT or A53T) and tau (WT or P301L) alone or in combination. W303-1A yeast strain was transformed with the integrative vectors pRS306 Gal1 harbouring ASYN (WT or A53T) and YIpLac128 Gal1 harbouring tau (WT or P301L) alone or in combination. Both transformation of yeast strains using the lithium acetate method and transformants colony selection were performed as described in [46]. Confirmation of integration was performed by PCR using the following primers: fw_aggagcacagacttagattg and rw_ttggagcctacatagagaac for the pRS306 Gal1 harbouring ASYN isoforms and fw_gcaatcgtcttactttctaa and rw_ gcaaatagtctacaaaccag for the YIpLac128 Gal1 harbouring tau isoforms.

### Spotting Assays and Growth Curves

Cell growth was analysed on solid selective SC medium to maintain selection for plasmids by spot assay by performing 5x-fold serial dilutions of exponential growing cultures, starting at OD_600_ = 1,0. For this, cells were pre-grown overnight at 30°C with agitation (200 rpm) in selective SC medium, containing raffinose. After this pre-incubation, cells were re-inoculated in the same selective SC medium at OD_600_ of 0,2 and left to grow at 30°C with agitation (200 rpm) until an OD_600_ of 1.0 was reached. Spot plates containing glucose (non-inducing medium) or galactose (inducing medium) were incubated at 30°C or 37°C with images being acquired after 72 h of incubation. Quantification analysis was performed using ImageJ [47] acquiring the intensity of all the spots in each lane from the plates containing galactose and then normalized versus the respective values obtained from the control plate containing glucose. Statistical significance was determined by performing one-way ANOVA with post-hoc Dunnett’s test using GraphPad Prism. Growth was also analysed in liquid medium. For this, cells were pre-grown overnight at 30°C with agitation (200 rpm) in selective SC medium containing raffinose. After this pre-incubation, cells were re-inoculated in selective SC medium containing glucose (non-inducing medium) or galactose (inducing medium) and incubated at 30°C or 37°C in 96 well plates. Growth was automatically monitorized by measuring OD_600_ using a PerkinElmer Victor 3V spectrophotometer.

### Immunoblotting

Immunoblotting was conducted following standard procedures [48] with some minor modifications. Yeast cells were pre-grown at 30°C with agitation (200 rpm) in selective SC medium containing raffinose. After this pre-incubation, cells were inoculated at OD_600_ of 0,2 in selective SC medium containing galactose and incubated overnight at 30°C (W303-1A) or 37°C (BY4741 WT and ΔRIM11) with agitation (200 rpm). Cells were harvested by centrifugation, washed in sterile water and pellets were resuspended in 100 µl of 1X SDS sample buffer (60 mM Tris-HCl pH 6.8, 10% glycerol, 2% SDS, 70 mM βME, 1% bromophenol blue supplemented with 1x protease inhibitor cocktail (Sigma-Aldrich) and 1x PhosSTOP (Roche) phosphatase inhibitor cocktail). After resuspension, pellets were lyzed by boiling for 5 minutes. 15 µl of each sample was run in a 12% SDS-PAGE gel and blotted onto PVDF membrane. Immunodetection was carried out using the following primary antibodies: Total tau (Polyclonal Rabbit anti-Human Tau, Dako) diluted 1∶10000, phospho tau in S396/404 (Mouse AD2 Anti-Tau Protein mAb, BioRad) diluted 1∶5000, ASYN (Purified Mouse Anti-α-Synuclein, BD) diluted 1∶1000 and GAPDH (Mouse Monoclonal Anti-GAPDH, Ambion) diluted 1∶3000, all in in TBST containing 1% milk. The following secondary antibodies were used: Goat Anti-Mouse IgG (H+L)-HRP Conjugate (BioRad) diluted 1∶10000 and oat Anti-Rabbit IgG (H+L), Horseradish Peroxidase conjugate (Invitrogen) diluted 1∶10000, all in TBST containing 1% milk. Membranes were revealed using Immobilon Western Chemiluminescent HRP Substrate (Millipore) and digital images acquired with Alliance 4.7 (UVITECH Cambridge). Quantification analysis was performed using ImageJ [47] and statistical significance was determined by performing one-way ANOVA with post-hoc Dunnett’s test using GraphPad Prism.

### Sarkosyl Fractionation

Sarkosyl fractionation method was performed as described in [49] with some minor modifications. Yeast cells were pre-grown overnight at 30°C with agitation (200 rpm) in selective SC medium containing raffinose. After this pre-incubation, cells were re-inoculated at OD_600_ = 0,2 in 50 ml of selective SC medium containing galactose and incubated for 24 hours at 30°C (for W303-1A) or 37°C (for BY4741 WT) with agitation (200 rpm). Cells were then harvested by centrifugation, washed in sterile water and pellets were resuspended in 500 µl of Extraction Buffer (100 mM Tris-HCl pH 7,9, 250 mM ammonium sulfate, 1 mM EDTA, 10% glycerol, 0,5 mM DTT supplemented with 1x protease inhibitor cocktail (Sigma-Aldrich)). Resuspended cells were homogenized with glass beads for 10 minutes at 4°C, total protein extract concentration was measured by Bradford protein measurement method assay [50] and adjusted to 1 mg/ml (Input). Sarkosyl (Sigma-Aldrich) was added to the lysates to a final concentration of 1% and samples were incubated at room temperature for 5 minutes. Sarkosyl-soluble and insoluble fractions were separated by centrifugation at 35,000 g for 1 h at 4°C. Pellets were washed once with extraction buffer and centrifuged at 35,000 g for 30 min to eliminate residual protein from soluble fractions. After centrifugation, pellets were resuspended in 15 µl of 1X SDS sample buffer and boiled for 5 min, before loading onto a 12% SDS-PAGE gel. Equal volumes of input and sarkosyl soluble fraction were diluted with 2X SDS sample buffer and boiled for 5 min, before loading onto 12% SDS-PAGE gel.

### Immunofluorescence

Immunofluorescence was performed as described in [51] with some minor modifications. Yeast cells were pre-grown at 30°C with agitation (200 rpm) in selective SC medium containing raffinose. After this pre-incubation, cells were re-inoculated at OD_600_ = 0,2 in 4 ml of selective SC medium containing galactose and incubated overnight at 30°C (W303-1A) or 37°C (BY4741 WT) with agitation (200 rpm). Cells were then fixed in 4% formaldehyde for 1 hour at 30°C. Cells were then harvested by centrifugation, washed in sterile water, treated to form spheroplasts, (1 hour at 30° in K_2_HPO_4_ 50 mM, KH_2_PO_4_ 50 mM, MgCl_2_ 0,5 mM, sorbitol 1,2 M, βME 70 mM, Lyticase 50 µg/ml, pH 6,8), washed in PBS and fixed onto poly-lysine-coated slides. Permeabilization was then performed with PBS containing 0,2% Triton-X100 for 15 minutes, after which cells were washed two times with PBS and subsequently blocked with PBS containing 0,5% BSA for 10 minutes. Fixed cells were then incubated with anti-ASYN primary antibody (ASYN Purified Mouse Anti-α-Synuclein, BD) diluted 1∶100 in PBS containing 0,1% BSA for 1 hour, washed and incubated with CY3 conjugated secondary antibody (Cy3® Goat Anti-Mouse IgG (H+L) Invitrogen) diluted 1∶500 in PBS containing 0,1% BSA for 30 minutes. After antibody labelling samples were mounted in a glycerol mounting media containing DAPI (1 mg/ml in 90% glycerol) and analysed using a Zeiss AxioObserver.D1 microscope. Image acquisition was performed with the software AxioVision (Zeiss). Statistical significance was determined by performing one-way ANOVA with post-hoc Dunnett’s test using GraphPad Prism.

## Results

### Cytotoxic Growth Effects Upon High-copy Plasmid Expression of ASYN Alone or in Combination with Tau

**Figure 1:**
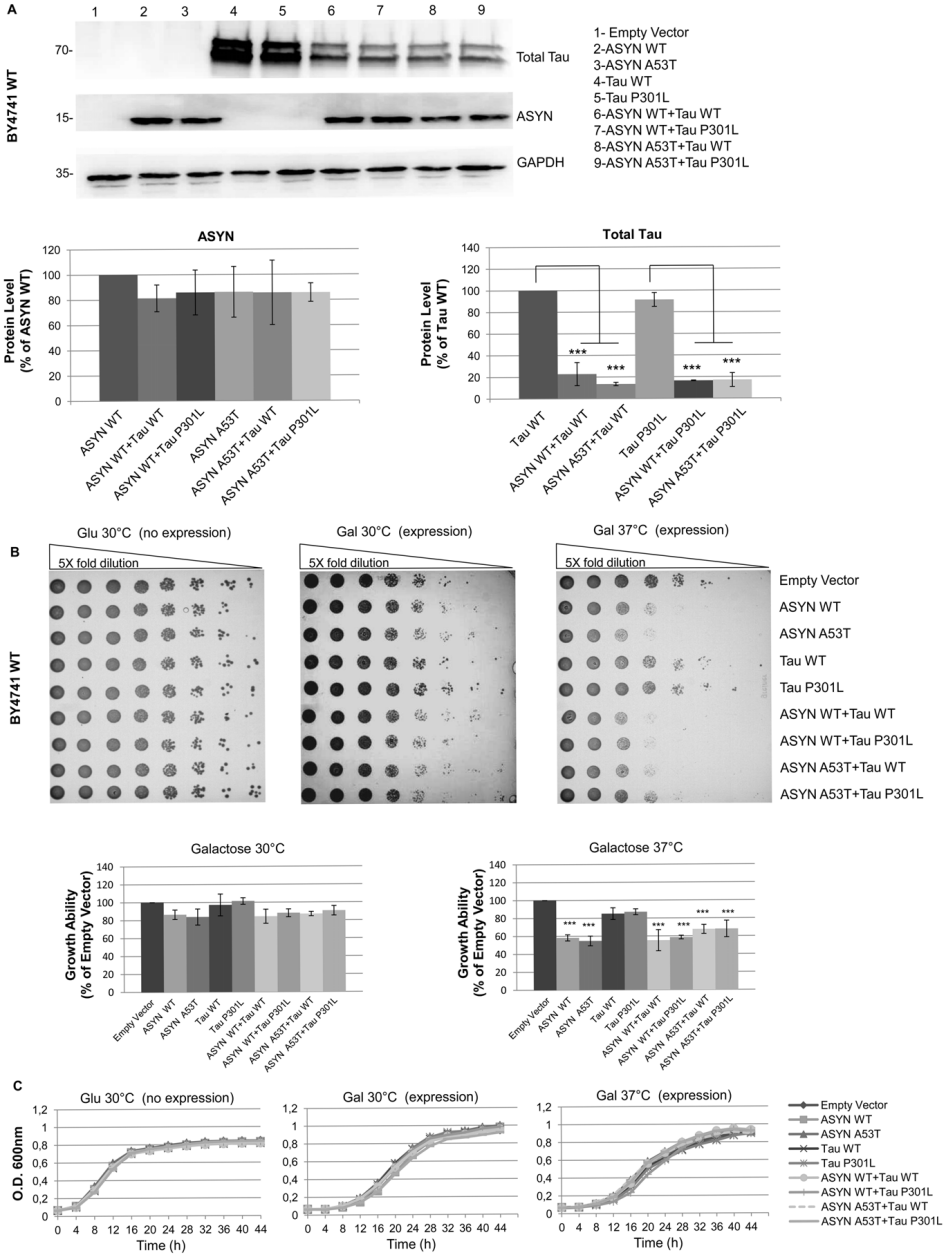
Cytotoxic growth effects upon high-copy plasmid expression of ASYN alone or in combination with Tau. A) High expression level of ASYN (WT and A53T) and tau (WT and P301L), detectable by western blot upon expression with pESC-LEU bidirectional high-copy (2µ) episomal vector. Whereas ASYN levels (WT and A53T) are similar when either expressed alone or in combination with tau (WT or P301L), tau (WT and P301L) levels are strongly decreased when expressed in combination with ASYN (WT or A53T), (***p<0,001). GAPDH was used as loading control. B) Strong growth delay observed by dot spot at 37°C after 72 hours of incubation upon expression of ASYN (WT and A53T) alone or in combination with tau (WT and P301L). Expression of tau (WT or P301L) alone has no effect on cells vitality whereas when expressed in combination with ASYN (WT and A53T) cytotoxicity is observed. A quantitative plot of the dot spots is shown. Results are representative of at least three independent experiments. C) No differences observed upon expression of ASYN and tau isoforms alone or in combination by growth ability tests performed in liquid media using 96 well plates at both 30°C and 37°C. All results are representative of at least three independent experiments.

We started promoting the co-expression of ASYN and tau 2N/4R isoforms in yeast by transforming the WT strain BY4741 with a bi-directional inducible high-copy plasmid containing ASYN (WT or A53T) and tau (WT or P301L) coding sequences alone or in combination. By using a bi-directional vector, our goal was to achieve a more controlled expression of both transgenes, as the use of two independent episomal plasmids results in copy number variation and, subsequently, in different and variable levels of protein expression. As observed by western blot, all the transformants showed equal protein expression levels of ASYN isoforms, while expression of tau isoforms strongly decreased when expressed in combination with ASYN (WT or A53T) (Fig. 1,A). This decrease is not a specific effect to ASYN, as co-expression of tau isoforms with a control protein led to the same alterations in tau total protein levels (data not shown). The growth profile upon expression of ASYN and tau variants alone and in combination was evaluated in both solid and liquid media. Dot spot analysis revealed that expression of ASYN (WT or A53T) at 37°C causes growth delay, as reported in previous studies [40]. In contrast, expression of tau (WT or P301L) had no effect on yeast fitness which has also been observed previously [38]. Yeast strains co-expressing ASYN isoforms and tau showed a similar growth delay as observed upon expression of ASYN alone, with no evident synergistic effect being detected. In contrast to the cytotoxic growth effect observed for ASYN (WT or A53T) expressed alone or in combination with tau (WT or P301L) in solid media, no growth delay was observed in liquid media (Fig. 1,C). These data strongly suggest that the cytotoxic growth effect phenotype observed in these episomal strains is mainly mediated by the expression of ASYN isoforms. Nevertheless, tau levels were reduced upon co-expression, which might have hindered the observation of a synergistic phenotype.

### ASYN Increases Tau Insoluble Aggregation State

**Figure 2:**
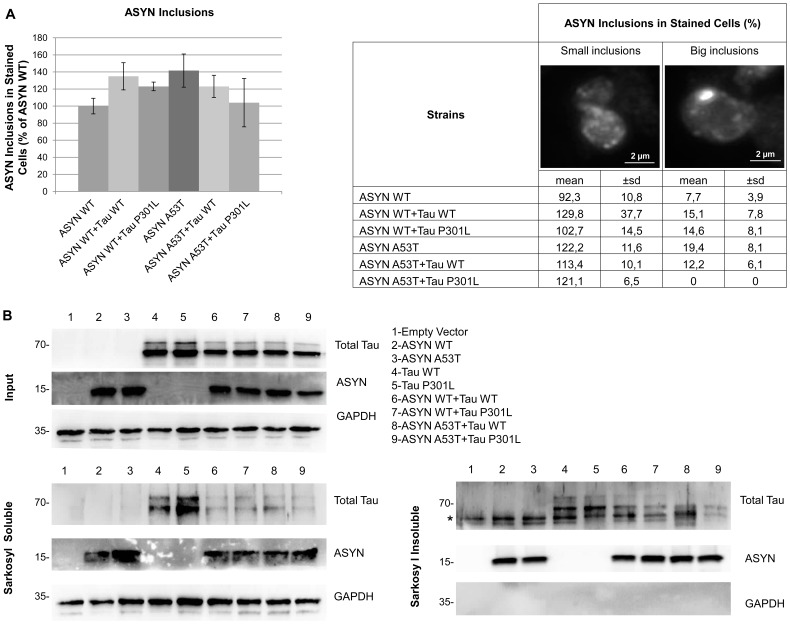
ASYN increases Tau insoluble aggregation state. A) Immunofluorescence with an anti-ASYN antibody showed no significant differences between the percentage of cells that contain ASYN (WT and A53T) intracellular inclusions when expressed alone or in combination with tau (WT and P301L). No yeast cells with ASYN big inclusions were observed in the strain expressing ASYN A53T in combination with tau P301L For statistical analysis at least 800 cells were counted. B) Both ASYN (WT and A53T) and tau (WT and P301L) form intracellular sarkosyl insoluble aggregates when expressed either alone or in combination, which are detectable by western blot. GAPDH was used as loading and soluble protein control. *corresponds to an unspecific band. Results are representative of three independent experiments.

It is demonstrated that in mammalian cellular models tau co-localizes with ASYN aggregates with overexpression of tau leading to an increase in the number of ASYN inclusions and to a reduction in their size [31], [52]. To further characterize and to assess if similar effects are present in the episomal yeast strains generated, the percentage of cells showing ASYN intracellular inclusions was quantified by immunofluorescence. No differences were observed, in terms of size and number of ASYN intracellular inclusions between the strains expressing ASYN isoforms alone or in combination with tau isoforms (Fig. 2,A). Interestingly, in the double mutant ASYN A53T/tau P301L, cells with large aggregates were not detected (Fig. 2,A). In order to quantify tau intracellular inclusions, anti-tau immunofluorescence was also tried using different antibodies but no specific signal was ever detected. As an alternative, sarkosyl fractionation was applied as this technique is largely used to characterize the solubility of intracellular protein aggregates in the brain of PD or AD patients [53], [54] as well as in animal and cellular models of neurodegeneration [43], [55], [56]. Results obtained showed that all ASYN and tau isoforms, expressed alone or in combination, are present in the sarkosyl insoluble fraction (Fig. 2,B). ASYN isoforms alone or in combination appear to be evenly distributed between the soluble and insoluble fractions. Tau WT seems to be slightly more concentrated in the insoluble fraction whereas tau P301L looks to be marginally more abundant in the soluble fraction (although this might be due to small differences in total protein loading, as GAPDH intensity also seems to be higher for this sample). Interestingly, co-expression of tau isoforms with ASYN leads to an increase in the proportion of insoluble tau (Fig. 2,B), as observed by the higher amount of tau present in the sarkosyl insoluble fraction when compared to the soluble fraction. Therefore, whereas tau expression, at least up to this protein level, does not seem to affect ASYN intracellular inclusion number and size, ASYN expression increases the insoluble fraction of tau.

### ASYN Increases Tau Phosphorylation in S396/404 via RIM11

**Figure 3:**
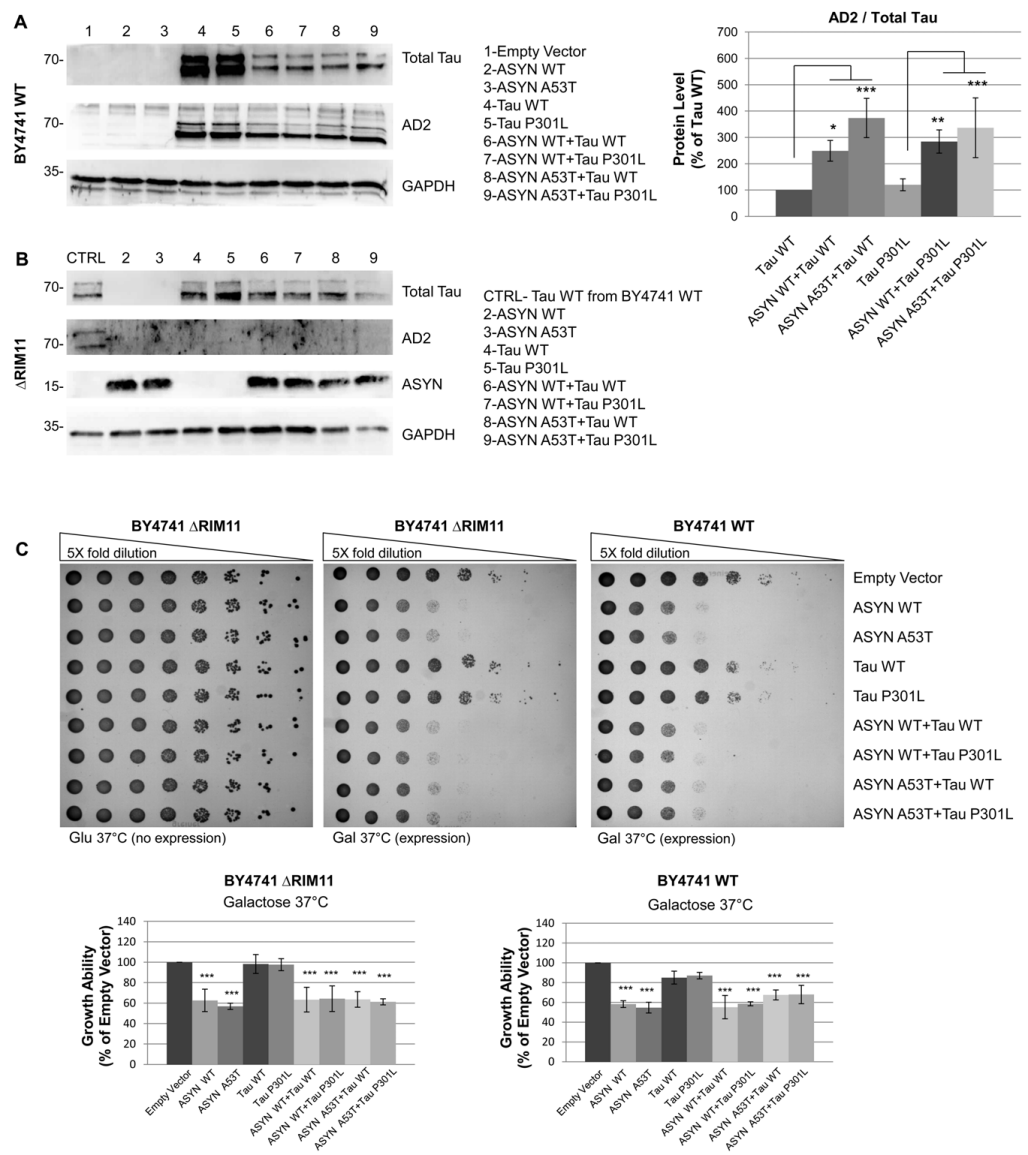
ASYN increases Tau phosphorylation in S396/404 via *RIM11*. A) Relative percentage of phosphorylated tau (WT and P301L) in S396/404 labelled by the antibody AD2 was increased in the presence of ASYN (WT and A53T). (*p<0,05; **p<0,01). GAPDH was used as loading control. B) No phosphorylated tau (WT or P301L) detected in ΔRIM11 mutant by western blot when expressed alone or in combination with ASYN (WT or A53T). GAPDH was used as loading control. C) Lack of tau (WT and P301L) phosphorylation in S396/404 doesn’t alter cytotoxicity observed in solid media when tau (WT or P301L) and ASYN (WT and A53T) are expressed alone or in combination in ΔRIM11 as compared to expression in BY4741 WT.A quantitative plot of the dot spots is shown (*p<0,05; **p<0,01; ***p<0,001). Results are representative of at least three independent experiments.

Hyperphosphorylated tau is the main component of PHF of NFT in the brain of AD patients [57], [58]. As we observed that the presence of ASYN leads to an increase in tau insoluble aggregation state (Fig. 2,B) we next evaluated the phosphorylation status of tau in the episomal yeast strain co-expressing ASYN and tau. To assess tau phosphorylation level, western blot analysis was performed using AD2 antibody, which recognizes phosphorylated tau at S396/404. This form of tau is crucial for tau fibrillization and [18], [19] is characteristic of PHF in Alzheimer’s disease [59]. Analysis of AD2 immunoreactivity versus total tau showed that tau WT and tau P301L are phosphorylated to the same extend when expressed alone (Fig. 3,A) but display a significant increase in S396/404 phosphorylation in the presence of ASYN. The increased relative level of phosphorylation of tau isoforms is even more striking upon co-expression with ASYN A53T (Fig. 3,A).

S306/404 of tau is a typical *GSK3β* substrate [20], [42]. In order to evaluate if the increased tau phosphorylation observed upon co-expression of ASYN was mediated by *GSK3β*, we used a yeast strain lacking the orthologue of human *GSK3β*. Yeast cells carry four orthologues of human *GSK3β* and it is already known that *RIM11* can act as a tau kinase and that the AD2 phosphoepitope is significantly reduced or absent in these mutant strains [42], [60]. Hence, the inducible high-copy plasmid expressing ASYN (WT or A53T) and tau (WT or P301L), alone or in combination, was transformed in a ΔRIM11 mutant yeast strain, in order to evaluate tau phosphorylation state in the presence and absence of ASYN. Results showed that the AD2 tau phosphoepitope was not detected in any of the samples tested, corroborating that the increased tau phosphorylation in S306/404 observed in the presence of ASYN is fully mediated by *RIM11*. Growth ability test in solid media showed that lacking of tau phosphorylation in S396/404 does not alter the growth phenotype observed in BY4741 WT yeast strain (Fig. 3,C). This was an expected outcome, as expression of tau seems to be non-related to the growth arrest effect observed. Taken together, the finding that tau phosphorylation in S396/404 via *RIM11* is increased in the presence of ASYN is in accordance with what has been described in other models, where it was demonstrated that *in vitro* ASYN directly stimulates tau phosphorylation via *GSK3β* [61], while *in vivo* and in post-mortem brain from PD high levels of active *GSK3β* and hyperphosphorylated tau were observed [62], [63]. Our yeast strain thus mimics relevant pathological features of neurodegeneration diseases where interaction between ASYN and tau occurs. Nevertheless, increased cytotoxicity due to tau hyperphosphorylation was not observed. This might be due to the lower tau intracellular levels upon co-expression with ASYN. A model where tau protein levels were higher would facilitate the assessment of pathological synergistic interactions with ASYN.

### Strong Synergistic Growth Effect Upon Genome Integration of ASYN WT and Tau WT

**Figure 4:**
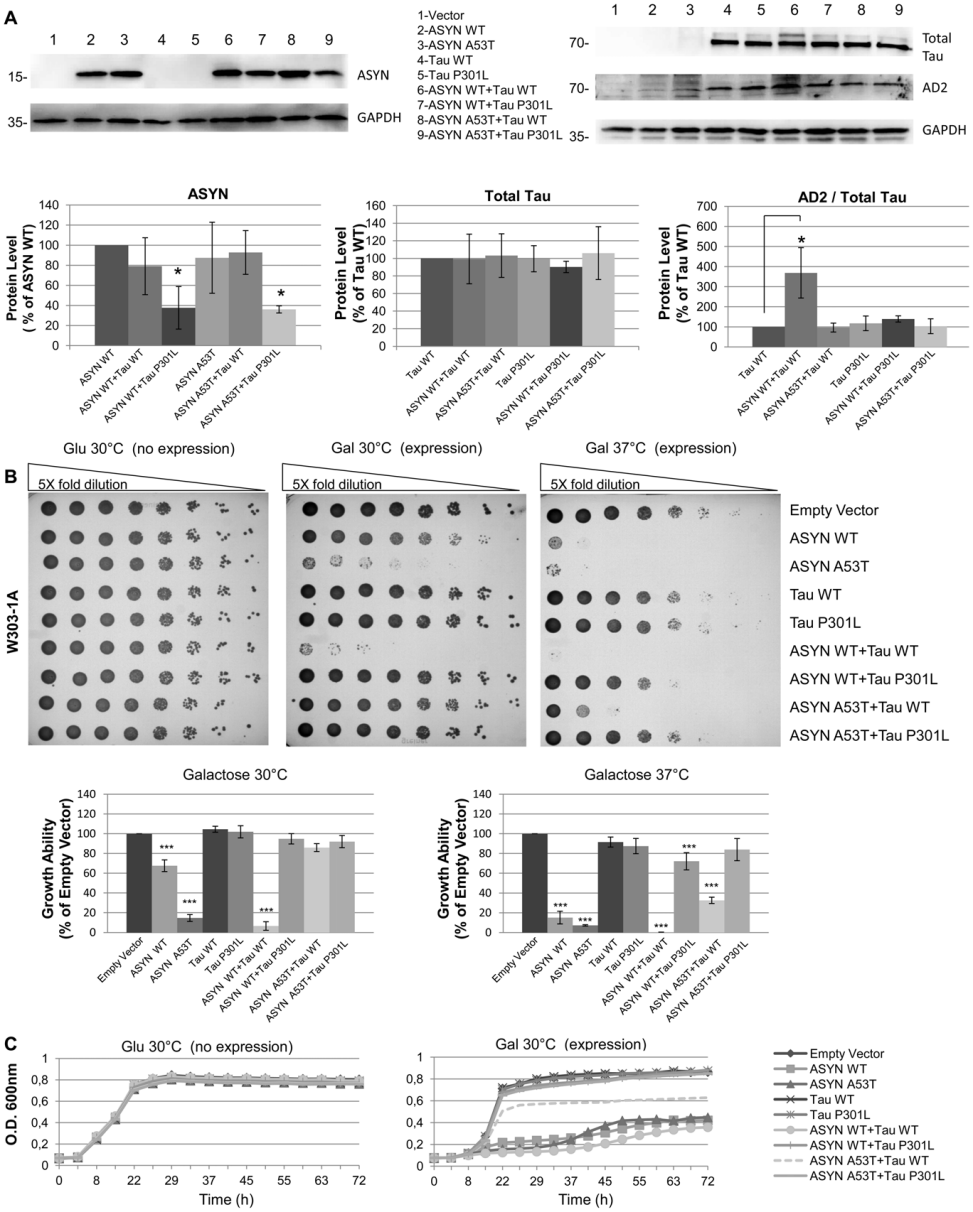
Strong synergistic growth effect upon genome integration of ASYN WT and Tau WT. A) ASYN protein expression levels detectable by western blot, remain generally constant, displaying a reduction upon co-expression of either tau isoform. Total tau expression levels remain constant whereas the percentage of tau WT phosphorylated in S396/404 increases only in the presence of ASYN WT. (*p<0,05; **p<0,01). GAPDH was used as loading control. B) Slight growth delay observed by dot spot at 30°C upon expression of ASYN WT, high growth delay with ASYN A53T and strong synergistic cytotoxicity observed when ASYN WT and tau WT are co-expressed. At 37°C a total growth arrest is observed for the strains expressing either ASYN (WT or A53T) alone or expressing ASYN WT in combination with tau WT. Tau P301L seems to rescue cytotoxic effect mediated by ASYN (WT and A53T) as well as tau WT when in combination with ASYN A53T. A quantitative plot of the dot spots is shown (*p<0,05; ***p<0,001). Results are representative of at least three independent experiments. C) Strong growth delay also observed in liquid growth analysis at 30°C upon co-expression of ASYN (WT and A53T) alone or ASYN WT in combination with tau WT. As in solid media tau P301L seems to rescue cytotoxic effect mediated by ASYN (WT and A53T) as well as tau WT when in combination with ASYN A53T. All results are representative of at least three independent experiments.

We next accessed the synergistic phenotype of ASYN and tau expression using an integrative yeast model by inserting one copy of ASYN (WT or A53T) and tau (WT or P301L), alone or in combination, into the genome of W303-1A yeast strain. Our goal was to use a more stable and independent system for the expression of the two transgenes, in order to achieve higher intracellular tau levels. Integration was performed using specific yeast vectors able to recombine with the yeast genomic DNA at defined sites and correct integration was confirmed by PCR. After generation of the integrative strains, ASYN and tau protein expression level and tau phosphorylation in S396/404 were evaluated by western blot. As desired, tau (WT and P301L) expression levels, alone and in combination with ASYN, were significantly higher and constant among all strains (Fig. 4,A). ASYN protein levels were generally constant, with the exception of strains co-expressing tau P301L, which displayed reduced expression of ASYN, for both WT and A53T isoforms (Fig. 4,A). The observed differences in ASYN protein levels can not be interpreted as a specific consequence of tau co-expression, as they might merely reflect clonal variation among strains. Interpretation of these differences needs further experimental clarification. Quantification of tau phosphorylation levels revealed that the presence of ASYN did not induce significant changes, most probably due to the lower levels of intracellular ASYN (or, at least, to the lower ratio of ASYN/tau) (Fig. 4,A). Exception to this was the strain co-expressing tau WT and ASYN WT, which displayed a huge increase in the relative fraction of phosphorylated tau.

Cytotoxic growth effects were also assessed in this integrative model in both solid and liquid media. As expected, dot spot results showed that expression of ASYN WT and A53T at 30°C caused moderate and high cytotoxic growth phenotypes, respectively (Fig. 4,B). Despite the high expression level of tau isoforms in this integrative model, tau expression alone didn’t show any effect in yeast growth in either solid or liquid media (Fig. 4,B and C). A strong synergistic growth effect was observed upon co-expression of ASYN WT and tau WT (Fig.4,B), which was even more pronounced at 37°C (Fig. 4,B). A similar growth delay was also obtained in liquid media showing the robustness of the alterations observed (Fig. 4,C). The synergistic growth effect observed for this integrative strain seems to be correlated with the increased tau phosphorylation state.

We observed an apparent rescue of toxic phenotype upon co-expression of ASYN (WT and A53T) with tau P301L, as the corresponding strains display increased growth fitness (Fig. 4, B). Nevertheless, expression levels of ASYN WT and ASYN A53T in both these strains is significantly lower (Fig. 4, A), and thus the improved growth might be merely a consequence of a less toxic intracellular environment due to less ASYN and not a true rescue by P301L.

### High Percentage of ASYN Cytoplasmatic Inclusions and Insoluble Tau Correlate with the Synergistic Growth Effect

**Figure 5:**
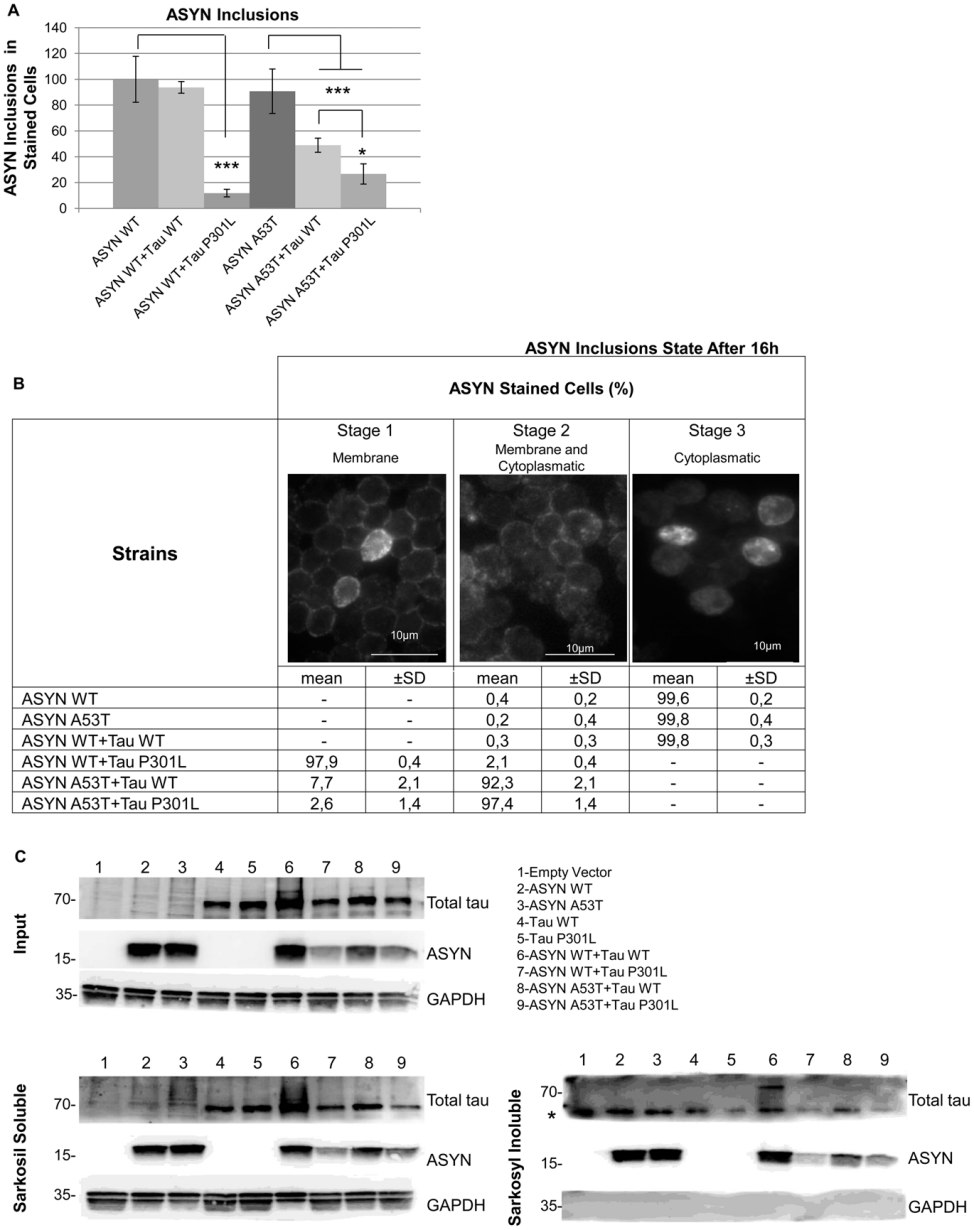
High percentage of ASYN cytoplasmatic inclusions and insoluble Tau correlate with the observed synergistic growth effect. A) Percentage of cells showing ASYN intracellular aggregates, detectable by immunofluorescence with anti-ASYN antibody, is similar when ASYN WT is expressed alone or in combination with tau WT, while when it is in combination with tau P301L it is highly reduced. Co-expression of ASYN A53T with tau (WT or P301L) also shows a decreased percentage of cells with intracellular aggregates. (*p<0,05; ***p<0,001). For statistical analysis at least 800 cells were counted. B) ASYN inclusions localization analyzed after 16 h, by immunofluorescence with anti-ASYN antibody, shows that in the strain expressing ASYN (WT or A53T) alone or ASYN WT in combination with tau WT inclusions are mainly cytoplasmatic. In the strains co-expressing ASYN A53T in combination with tau (WT or P301L) both small membrane associated and cytoplasmatic inclusions are detectable, whereas only small membrane associated inclusions can be observed when ASYN WT is expressed in combination with tau P301L For statistical analysis at least 800 cells were counted. C) ASYN (WT and A53T) alone or in combination with tau (WT or P301L) is detectable by western blot in the sarkosyl insoluble fraction, whereas tau is detectable in the sarkosyl insoluble fraction only when co-expressed with ASYN WT. GAPDH was used as loading and soluble protein control. *corresponds to an unspecific band. Results are representative of at least three independent experiments.

To further investigate the synergistic interaction detected between ASYN and tau in the integrative yeast strains developed in this work, we accessed the aggregation state of ASYN and tau by immunofluorescence and sarkosyl fractionation experiments. Interestingly, the number of ASYN intracellular inclusions detected by immunofluorescence didn’t remain constant in all strains. Strains expressing ASYN alone or ASYN WT in combination with tau WT displayed a high level of cells with ASYN inclusions. A reduction in the number of cells with inclusions was observed in the strain expressing ASYN WT in combination with tau P301L and in both the strains expressing ASYN A53T in combination with tau (WT or P301L) (Fig. 5,A). Furthermore, changes in ASYN intracellular inclusion localization were also observed. It is known that in yeast ASYN initially localizes to the membrane, and subsequently starts to form small membrane localized inclusions that finally convert into larger cytoplasmatic inclusions [44]. The majority of yeast cells expressing ASYN isoforms alone and ASYN WT in combination with tau WT showed only the most advanced cytoplasmatic inclusions. On the other hand, the strain co-expressing ASYN WT and tau P301L showed only small membrane connected inclusions, whereas in the strains co-expressing ASYN A53T and tau isoforms both membrane connected and cytoplasmatic inclusions were observed (Fig. 5,B). Sarkosyl fractionation assays showed ASYN equally distributed between the sarkosyl soluble and insoluble fractions in all the strains expressing ASYN isoforms alone or in combination with tau isoforms, whereas insoluble tau WT was only detected when co-expressed with ASYN WT (Fig. 5,C). These findings suggest that the synergistic growth effect observed upon co-expression of ASYN WT and tau WT in this integrative model is probably triggered by the high percentage of ASYN cytoplasmatic inclusions and by the increased fraction of insoluble tau.

## Discussion

Interactions between ASYN and tau have been shown to occur in several neurodegenerative diseases, as relevant as AD and PD. Although some light has been shed on the cellular effects resulting from this interaction [64], a full understanding of the cellular mechanisms triggered by this toxic duet has not been achieved yet. In this work two different yeast sets of strains were developed and characterized with the final aim of being used as tools for the discovery of potential target genes that mediate the interaction between ASYN and tau.

We started by promoting the expression of ASYN and tau isoforms in yeast from a bidirectional episomal vector. Expression of ASYN isoforms alone led to a cytotoxic growth phenotype, as reported before [40]. On the contrary, no changes in yeast growth properties were observed upon expression of tau WT or mutants, also in accordance with previous observations [43]. In our study, co-expression of ASYN and tau from a bidirectional episomal vector resulted in no evident synergistic toxic effect. Previous data showed that co-expression of tau and ASYN in yeast resulted in synergistic growth delay [44], although in that case, no cytotoxicity upon expression of ASYN alone was observed, in clear contrast to the majority of the published studies.

In our episomal co-expression strains, ASYN levels remained constant in all the strains analysed, whereas tau levels strongly decreased upon co-expression with ASYN. The observed reduction in tau protein levels is not specific to the co-expression of ASYN. Similar results were observed upon co-expression of tau and other proteins and most probably reflect a lower protein expression efficiency resulting from the simultaneous use of the Gal1-Gal10 divergent promoter and subsequent downstream processes. ASYN levels were not affected by co-expression of a second transgene, which might be due to the small size of this transcript, which allows higher protein expression efficiency. Overall, co-expression of ASYN and tau had no evident effect on the size and number of ASYN intracellular inclusions.

Our results showed that the presence of ASYN affected the solubility of tau, increasing the fraction of insoluble/aggregated protein, both for tau WT and tau P301L. To further explore this evidence we analysed tau phosphorylation at Ser396/404, as tau phosphorylation is known to lead to the formation of insoluble tau aggregates. Indeed, co-expression of ASYN isoforms with either tau WT or tau P301L led to a significant increase in tau phosphorylation, particularly in the case of co-expression of tau isoforms with ASYN A53T. ASYN has been reported to directly stimulate tau phosphorylation by *GSK3β* by making part of a heterotrimeric complex containing ASYN, tau and *GSK3β* [30], [61]. The pathological epitope Ser396/404 is a typical *GSK3β* substrate, already shown to be a target for tau phosphorylation in yeast [42]. We showed that in our model the presence of ASYN led to increased aggregation of tau through phosphorylation at Ser396/404 via *RIM11*, the yeast orthologue of *GSK3β*. In RIM11-deleted strain, no phosphorylation at Ser396/404 was observed. To our knowledge, this is the first evidence that ASYN is able to induce tau phosphorylation and aggregation in yeast, proving that yeast recapitulates the reported mechanism through which ASYN stimulates *GSK3β*, leading to phosphorylation of tau at pathogenic epitopes [61].

Despite the increased phosphorylation and aggregation of tau WT and tau P301L promoted by co-expression of ASYN isoforms, no synergistic differences in growth phenotype were detected due to alterations in phosphorylated tau in our episomal yeast strain. Nevertheless, previous studies in yeast have consistently revealed that tau expression *per se* does not affect growth, despite tau phosphorylation, conformational changes and accumulation into aggregates [38]. In addition, the precise nature of the toxic tau species and the exact sequence of events leading to tau-mediated toxicity are not consensual. Several lines of evidence suggest that tau aggregates are not the main cause of cell death, as neuron loss can be observed in the absence of tau tangles in both Drosophila and mice models [65]–[67] and other mice models show dissociation between brain areas of neurofibrillary tangles formation and neuronal loss [68], [69]. Results obtained with our episomal strain therefore mimic a relevant feature of human neurodegeneration, although not resulting in a measurable synergistic phenotype of growth arrest. Different observations were previously described for ASYN and tau co-expression in yeast [44]. Nevertheless, the authors used a different approach, expressing each protein from an independent episomal plasmid (and thus not allowing for copy number control and stability). Additionally, tau protein levels and phosphorylation state were not accessed in the reported study, thus not allowing a more direct comparison with the results obtained in the present work.

We next evaluated the co-expression of ASYN and tau isoforms in yeast by making use of a different experimental approach, based on the stable integration of one copy of each transgene in the yeast genome. Our main goal was to achieve higher tau expression levels, in order to maximize any eventual underlying phenotypic change. Using this integrative approach, tau protein levels were found to be constant in all the generated strains, whereas ASYN levels showed variations. Again, expression of ASYN isoforms alone caused cytotoxic growth delay, more pronounced in the case of ASYN A53T. Despite the high and constant expression levels, the presence of tau isoforms alone had no implication in yeast growth phenotype.

Yeast strains co-expressing tau P301L with either ASYN isoform seemed to display a milder phenotype than strains expressing ASYN isoforms alone. Nevertheless, this “rescue” is only apparent has it reflects the low levels of ASYN in the yeast strains co-expressing tau P301L. Accordingly, strains co-expressing ASYN isoforms with tau P301L display a reduced number of ASYN inclusions when compared to the strains expressing ASYN WT or A53T alone. Moreover, these inclusions were predominantly located at the cell membrane, indicating an early stage of toxicity [40], as no cytoplasmatic ASYN aggregates were observed.

A strong synergistic effect in yeast growth was observed upon stable integration and co-expression of ASYN WT and tau WT. Similar observations were previously reported for the co-expression of these two proteins from two episomal vectors in yeast [44], although the typical growth delay upon expression of ASYN alone did not occur. The synergistic phenotype observed in our study was associated with increased phosphorylation of tau at Ser396/404, presumably via *RIM11* as a consequence of stimulation by ASYN. High levels of ASYN intracellular inclusions were present in this strain, all located in the cytoplasm and thus representative of late aggregation stages [40]. Most importantly, the yeast strain expressing ASYN WT and tau WT also showed to display an increased fraction of insoluble tau. It is known that ASYN toxicity in yeast is directly correlated to aggregation and inclusion formation [40], [70] and the number of cells which displayed ASYN inclusions, as well as the localization of those inclusions, were identical between the strain expressing ASYN WT and the strain co-expressing ASYN WT and tau WT. As no differences in the pattern of cytoplasmatic inclusions were registered, the increased toxic phenotype observed must be triggered by the high intracellular levels of both proteins, leading to the concomitant presence of a high number ASYN inclusions and a high amount of phosphorylated and aggregated tau. Occurrence of a measurable synergistic growth arrest phenotype was also favoured by the use of the W303-1A yeast strain, whose genetic background is more sensitive than the BY4741 yeast strain. With W303-1A, evident growth delay upon expression of ASYN alone can be observed already at 30°C (Fig. 4,B) whereas for BY4741 only at 37°C the same phenotype is achieved (Fig. 1, B). Three mutations differentiate W303-1A from BY4741: 1) Ybp1-1 mutation which abolishes *Ybp1* function, increasing sensitivity to oxidative stress, 2) Rad5-G535R missense mutation which abolishes *Rad5* function, involved in DNA repair and 3) *Bud4* affecting axial budding [71]–[73]. The use of a genetically more sensitive strain, together with the high levels of ASYN inclusion formation and tau phosphorylation and aggregation favoured the occurrence of a measurable synergistic cytotoxic phenotype [74].

In summary, our findings demonstrated that for the yeast strains developed, ASYN directly induced tau phosphorylation in S396/404 via *RIM11* and that formation of tau insoluble aggregates seemed to be dependent on tau phosphorylation level. Expression of ASYN, but not tau alone, led to growth retardation and a strong synergistic growth effect was observed upon genome integration of ASYN WT and tau WT. Therefore, we have developed yeast strains that recapitulate many of the most relevant aspects of ASYN and tau interactions in human pathology. The choice of yeast as the model organism will allow easy genetic manipulation for the identification of genes that can modulate the interaction between ASYN and tau. The episomal and integrative yeast strains presented here are powerful tools for rapid genome-wide analysis, allowing the combined development of both yeast knockout and plasmid overexpression screenings. In particular, the integrative strain displaying a synergistic toxic phenotype due to the co-expression of ASYN and tau is the model of choice for conducting screens in the search for genes that can be future targets for the development of new therapies for neurodegenerative diseases.
